# Improved Survival of Lymphoma Patients with COVID-19 in the Modern Treatment and Vaccination Era

**DOI:** 10.3390/cancers14174252

**Published:** 2022-08-31

**Authors:** Alexandra Della Pia, Charles Zhao, Parul Jandir, Amolika Gupta, Mark Batistick, Gee Youn (Geeny) Kim, Yi Xia, Jaeil Ahn, Gabriella Magarelli, Brittany Lukasik, Lori A. Leslie, Andre H. Goy, Andrew Ip, Tatyana A. Feldman

**Affiliations:** 1 Hackensack University Medical Center, Hackensack, NJ 07601, USA; 2 Ernest Mario School of Pharmacy, Rutgers University, Piscataway, NJ 08854, USA; 3 Hackensack Meridian School of Medicine, Nutley, NJ 07110, USA; 4 Department of Biostatistics, Bioninformatics, and Biomathematics, Georgetown University, Washington, DC 20057, USA; 5 John Theurer Cancer Center, Hackensack University Medical Center, Hackensack, NJ 07601, USA

**Keywords:** lymphoma, COVID-19, omicron, monoclonal antibodies, vaccines

## Abstract

**Simple Summary:**

Patients with lymphoma are at greater risk of complications from COVID-19 infection. However, limited data exists on COVID-19-related outcomes in lymphoma patients since the use of COVID-19 vaccines and treatments began. Our study reports the real-world outcomes of 68 lymphoma or CLL patients who developed COVID-19 infection during the omicron surge in the US. We found that 34% of patients were hospitalized due to COVID-19 infection. The COVID-19-associated death rate was 9% (6/68) in all patients and 26% (6/23) in hospitalized patients, which was much lower compared to rates earlier in the pandemic prior to the introduction of COVID-19 vaccines and treatments. In 30 patients with data available, 60% did not make antibodies after COVID-19 vaccination. Most patients (74%, 17/23) who were hospitalized did not receive COVID-19 monoclonal antibody treatment. Our results pointed to important differences and the need for a new approach to treating cancer patients with COVID-19 infection.

**Abstract:**

Lymphoma patients are at greater risk of severe consequences from COVID-19 infection, yet most reports of COVID-19-associated outcomes were published before the advent of COVID-19 vaccinations and monoclonal antibodies (mAbs). In this retrospective study, we report the real-world outcomes of 68 lymphoma or CLL patients who developed COVID-19 infection during the omicron surge in the US. We found that 34% of patients were hospitalized as a result of COVID-19 infection. The death rate due to COVID-19 was 9% (6/68) in the overall population and 26% (6/23) in hospitalized patients. During the preintervention COVID-19 era, the mortality rate reported in cancer patients was 34%, which increased to 60.2% in hospitalized patients. Thus, the death rates in our study were much lower when compared to those in cancer patients earlier in the pandemic, and may be attributed to modern interventions. In our study, 60% (18/30) of patients with serology data available did not develop anti-COVID-19 spike protein antibodies following vaccination. Most patients (74%, 17/23) who were hospitalized due to COVID-19 infection did not receive COVID-19 mAb treatment. Our results pointed to the importance of humoral immunity and the protective effect of COVID-19 mAbs in improving outcomes in lymphoma patients.

## 1. Introduction

Patients with malignancies are at an increased risk of severe morbidity and mortality from severe coronavirus disease 2019 (COVID-19) infection [1,2]. Unique challenges across patients with varying cancer types include the receipt of immunosuppressive therapies that blunt the immune response and diminish the ability to fight infection. Of patients with malignancies, lymphoma patients are comparatively at greater risk of severe consequences from COVID-19 infection. Lymphomas are a heterogeneous group of hematologic malignancies that is broadly divided histologically into Hodgkin’s lymphoma (HL) and non-Hodgkin’s lymphomas (NHLs), which comprise cancers of B-cell, T-cell, and natural killer (NK)-cell origin. The disease biology of lymphoma is associated with dysfunction of the innate and adaptive immune systems, which translates to impaired production of neutralizing antibodies in patients infected with COVID-19. In addition to the intrinsic immune dysfunction accompanied by the disease burden, anticancer agents such as anti-CD20 monoclonal antibodies and Bruton’s tyrosine kinase inhibitors (BTKis), which are used to treat lymphomas, may further deplete the host humoral response. Taken together, this leads to lower rates of seroconversion following vaccination or natural infection and increased risk of COVID-19-related serious events such as ICU admission, mechanical ventilation support, or death [3].

Advancements have been made in the management of patients with COVID-19 infection to prevent progression to severe disease and death. At the onset of the pandemic, convalescent plasma infusions from donors who recovered from COVID-19 infection were utilized to provide passive immunity to patients who developed severe COVID-19 infection. Studies with convalescent plasma in COVID-19 patients have shown conflicting results, likely due to differences in neutralizing antibody titers of preparations, timing of administration in the course of infection, and exclusion of immunocompromised patients [4]. In addition to passive immunity, research efforts against COVID-19 focused on therapies that either inhibited viral replication or modulate humoral immunity. In April 2020, the first trial evaluating the use of the antiviral agent remdesivir showed that patients with severe COVID-19 infection who received remdesivir had a numerically faster time to clinical improvement than those who received a placebo, with symptom duration of 10 days or less (hazard ratio (HR) 1.52; 95% CI, 0.95–2.43) [5]. In the next few months, trials that were conducted to evaluate the safety and efficacy of the COVID-19 vaccines found the mRNA vaccines to be 94–95% effective in the general population [6,7]. Additionally, trials that evaluated the efficacy of mAb therapy in outpatients who were at risk of developing severe COVID-19 infection found a significantly reduced risk of COVID-19-related hospitalization or death [8,9,10]. Although these therapies have shown significant benefit in the general population, these studies excluded patients with malignancies and lacked consideration of differences in the disease course of COVID-19 infection in immunocompromised patients. 

In November 2021, the omicron (B.1.1.529) variant emerged as a new COVID-19 variant of concern (VOC) due to its rapid spread and heavily mutated spike protein, which could potentially render antibody responses generated by COVID-19 infection or vaccination ineffective [11]. At this time, there was growing concern for the efficacy of COVID-19 mAb treatments against this new variant, as omicron carries over 30 mutations in its COVID-19 spike protein [3]. While earlier mAb combinations (bamlanivimab/etesevimab and casirivimab/imdevimab) were found to have decreased allorecognition against the novel omicron variant, others such as sotrovimab, bebtelovimab, and tixagevimab/cilgavimab (Evusheld) were shown to retain neutralization activity against omicron [12]. In cancer patients, the omicron variant is of great concern due to the increased risks of serious COVID-19-related outcomes. In particular, lymphoma patients are at a substantial risk of poor outcomes from COVID-19 infection due to immune system dysfunction and receipt of immunosuppressive therapies. While studies showed that there may be decreased humoral immunity but preserved T-cell immunity against the omicron variant, the efficacy of cellular immunity acquired from previous COVID-19 infection or vaccination against the omicron variant in lymphoma patients is unknown [13].

Most reports of COVID-19-associated morbidity and mortality were published before the advent of COVID-19 vaccinations and mAb treatments targeted against the spike protein. Prospective data on the efficacy of COVID-19 mAbs in immunocompromised patients are lacking. Here, we report the real-world outcomes of lymphoma patients who developed COVID-19 infection during the omicron surge in the United States (US).

## 2. Materials and Methods

This was a retrospective, single-center study at the John Theurer Cancer Center (JTCC) within Hackensack Meridian *Health* (HMH). Eligible patients were ≥18 years, had a diagnosis of lymphoma or chronic lymphocytic leukemia (CLL), and had developed COVID-19 infection between 1 December 2021 and 31 January 2022 during the omicron surge in the US. COVID-19 infection was defined as a positive result on PCR assay on or after 1 December 2021. Data were manually extracted by the team from HMH’s electronic health record system (Epic). Types of data collected included patient demographics, clinical characteristics, anticancer treatments, and COVID-related treatments and outcomes. Quality control was performed by physicians overseeing the data collection.

Institutional Review Board (IRB) approval was obtained from HMH. The trial was conducted under the International Conference on Harmonization Good Clinical Practice guidelines and according to the Declaration of Helsinki. The requirement for patient informed consent (verbal or written) was waived by the IRB because this project represented a noninterventional study utilizing routinely collected data for secondary research purposes.

The primary endpoint was COVID-19-associated hospitalization or death. Secondary endpoints included COVID-19-associated hospitalization or death in the following patient subgroups: different lymphoma subtypes, COVID-19 vaccination status, receipt of COVID-19 mAb treatment, patients who received anti-CD20 monoclonal antibody therapy within the previous 12 months, and patients who were receiving active anticancer treatment. Patients were further described according to lymphoma subtypes, which included the following groups: chronic lymphocytic leukemia (CLL)/small lymphocytic leukemia (SLL), Burkitt’s lymphoma/diffuse large B-cell lymphoma (DLBCL)/primary mediastinal B-cell lymphoma (PMBL), indolent B-cell lymphomas, mantle cell lymphoma (MCL), Hodgkin’s lymphoma/T-cell lymphomas (HL/TCLs), and other lymphoma types. Hodgkin’s lymphoma and T-cell lymphomas (HL/TCLs) were grouped together in the analysis because these patients did not receive anti-CD20 monoclonal-antibody-containing regimens and due to low patient numbers. Indolent lymphomas were defined as having a diagnosis of one of the following: follicular lymphoma (FL), marginal zone lymphoma (MZL), lymphoplasmacytic lymphoma, or Waldenstrom macroglobulinemia. T-cell lymphomas were defined as having a diagnosis of one of the following: peripheral T-cell lymphomas not otherwise specified (NOS), anaplastic large cell lymphoma (ALCL), cutaneous T-cell lymphomas, angioimmunoblastic T-cell lymphoma (AITL), and acute T-cell leukemia/lymphoma (ATLL). Patients were considered to have received active anticancer treatment if any therapy intended to treat cancer was administered within the six months prior to COVID-19 infection. Additional secondary outcomes included hospital length of stay and intensive care unit (ICU) admission.

Descriptive statistics were used to summarize the demographic and clinical characteristics. The outcome of COVID-19-associated hospitalization or death was compared among subgroups of interest using Fisher’s exact test or Pearson’s chi-squared tests, and univariate and multivariable logistic regression models were then fitted with corresponding odds ratios (OR) and confidence intervals (CI). A two-sided *p*-value < 0.05 was deemed statistically significant; all analyses were performed using R (*ver* 4.1, R Core Team (2022), Vienna, Austria) software.

## 3. Results

### 3.1. Patient Characteristics

Sixty-eight lymphoma patients were identified who tested positive for COVID-19 infection (Table 1). Baseline characteristics were as follows: median age of 66 years (IQR 59–75), 56% of patients were male, and 66% of patients were white. The most frequent comorbidities included cardiovascular disease (34%), history of smoking (31%), and diabetes (28%). Lymphoma subtypes included 20 (29%) patients with DLBCL/Burkitt’s/PMBL, 20 (29%) patients with indolent lymphomas, 14 (21%) patients with CLL/SLL, 6 (10%) patients with T-cell lymphomas, 3 (4%) patients with MCL, 2 (3%) patients with HL, and 3 (4%) patients with other lymphoma types. Fifty-three patients (78%) were vaccinated against COVID-19, with most patients (98%, 52/53) having received one of the COVID-19 mRNA vaccines.

### 3.2. Primary Outcome

**Figure 1:**
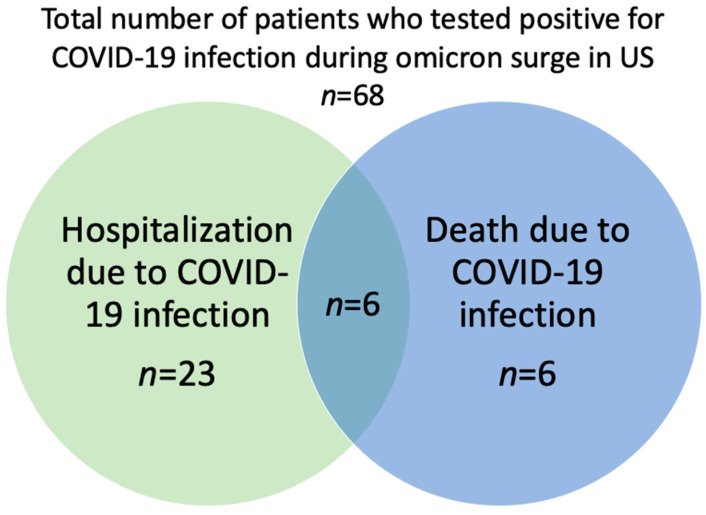
COVID-19-associated hospitalization or death in the overall population.

During the omicron surge in the US, 34% (23/68) of the patients were hospitalized due to COVID-19 infection (Table 2, Figure 1). The death rate due to COVID-19 was 9% (6/68) in the overall population, whereas the mortality in hospitalized patients was 26% (6/23) (Table 2, Figure 1). The median hospital length of stay (LOS) was 7 days (IQR 2-18) and two patients required admission to the ICU. There were six (9%) deaths from COVID-19 infection: one CLL patient receiving acalabrutinib; one TCL patient who was severely immunocompromised due to a bone marrow transplant; one DLBCL patient with refractory lymphoma enrolled in a clinical trial with bi-specific T-cell engager (BiTE) therapy; one TCL patient receiving hyperCVAD (hyper-fractionated cyclophosphamide, vincristine, doxorubicin, and dexamethasone) chemotherapy; one marginal zone lymphoma (MZL) patient receiving R-CHOP (rituximab, cyclophosphamide, doxorubicin, vincristine, and prednisone) chemotherapy; and one DLBCL patient with central nervous system (CNS) involvement receiving high-dose methotrexate and ibrutinib therapy. Most expired patients (5/6) did not receive COVID-19 mAb treatment.

### 3.3. Secondary Outcomes

#### 3.3.1. COVID-19-Related Outcomes in Patients Who Were Vaccinated against COVID-19

**Figure 2:**
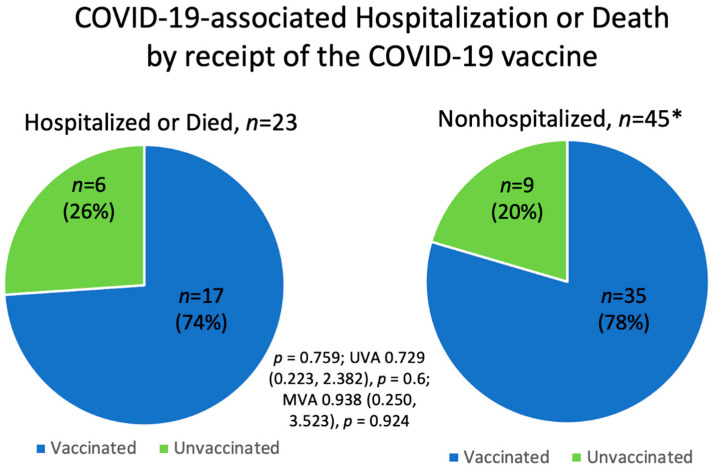
COVID-19-associated hospitalization or death according to receipt of the COVID-19 vaccine. * One patient with unknown vaccination status.

The majority of patients (76%, 52/68) in this study were vaccinated against COVID-19, with 74% (17/23) of hospitalized patients and 78% (35/45) of nonhospitalized patients having completed at least the initial COVID-19 vaccination series. There was no significant difference in COVID-19-associated hospitalization or death between vaccinated and unvaccinated patients (*p* = 0.759; UVA OR 0.729 (0.223, 2.382), *p* = 0.6; MVA OR 0.929 (0.222, 3.886), *p* = 0.919) (Figure 2, Table 3). Of the 52 patients who received the COVID-19 vaccine, 31% (16/52) were hospitalized and 4% (2/52) were escalated to ICU care. There were four patients (4/52, 8%) who died despite receiving the COVID-19 vaccine: one CLL patient receiving acalabrutinib; one TCL patient who was severely immunocompromised due to bone marrow transplant; one TCL patient receiving hyperCVAD (hyper-fractionated cyclophosphamide, vincristine, doxorubicin, and dexamethasone) chemotherapy; and one MZL patient receiving R-CHOP (rituximab, cyclophosphamide, doxorubicin, vincristine, and prednisone) chemotherapy.

Serology data following COVID-19 vaccination were available in 30 patients (58%, 30/52). Of these 30 patients, 40% (12/30) were positive for anti-COVID-19 spike protein antibodies following vaccination and all (100%, 12/12) remained nonhospitalized during COVID-19 infection. There were 18 patients (60%, 18/30) who did not seroconvert following COVID-19 vaccination and with resultant serology data available. Of these 18 patients, 33% (6/18) were hospitalized during COVID-19 infection and 67% (12/18) were nonhospitalized. Of the 23 patients who were hospitalized or died due to COVID-19 infection, serology data were available in 6 patients who were hospitalized and in 2 patients who died; all (100%, 8/8) of these patients were seronegative for anti-COVID-19 spike protein antibodies following COVID-19 vaccination.

#### 3.3.2. COVID-19-Related Outcomes in Patients Who Received COVID-19 Monoclonal Antibody Treatment

**Figure 3:**
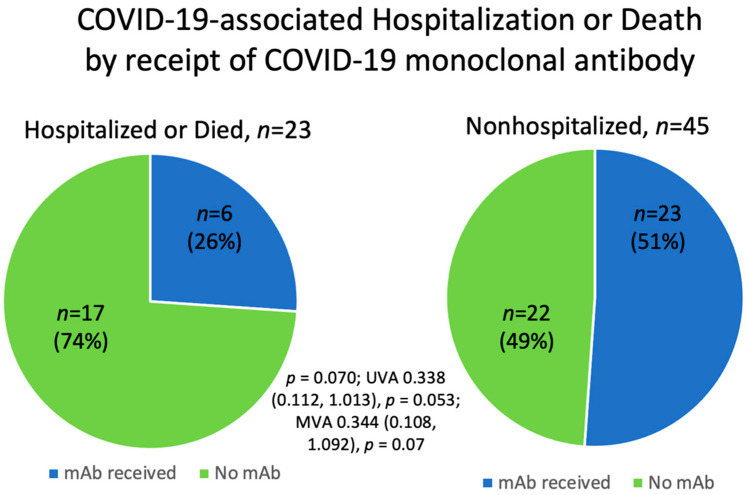
COVID-19-associated hospitalization or death according to receipt of COVID-19 monoclonal antibody treatment.

Most patients (74%, 17/23) who were hospitalized or died due to COVID-19 infection did not receive COVID-19 mAb treatment. Differently, 26% (6/23) of patients who were hospitalized or died received COVID-19 mAb treatment, although this was not statistically significant (*p* = 0.070; UVA OR 0.338 (0.112, 1.013), *p* = 0.053; MVA OR 0.324 (0.096, 1.098), *p* = 0.07) (Table 3, Figure 3). Of the 29 patients who received COVID-19 mAb therapy, 21% (6/29) were hospitalized and 3 (3/6, 50%) of these patients had a hospital LOS of 1 day, with none (0/6) of the patients having been escalated to ICU care. There was one patient (1/29, 3%) who died despite receiving COVID-19 mAb treatment; this was a MZL patient who recently received R-CHOP (rituximab, cyclophosphamide, doxorubicin, vincristine, and prednisone) chemotherapy.

#### 3.3.3. COVID-19-Related Outcomes in Additional Patient Subgroups

Patients who received anti-CD20 monoclonal antibody treatment or BTKi-containing regimens did not have significantly different rates of COVID-19-related hospitalization or death when compared to those who did not (*p* = 0.907) (Table 3). Additional subgroups of interest included lymphoma subtypes and those who received anticancer treatment in the previous 6 months; however, there was no significant difference in the primary outcome when these patient groups were considered (Table 3). At the time of COVID-19 diagnosis, there were six patients with an absolute neutrophil count less than 500 cells/mm^3^. Of these six patients, 83% (5/6) were hospitalized, none were admitted to the ICU, two patients received COVID-19 mAb treatment, and none died. Similarly, there were 15 patients with an absolute lymphocyte count (ALC) less than 1000 cells/mm^3^. Of these 15 patients, 60% (9/15) were hospitalized, none were admitted to the ICU, 8 patients received COVID-19 mAb treatment, and 2 patients died.

## 4. Discussion

In this single-center retrospective study, we found that 34% of patients with lymphoma or CLL were hospitalized as a result of COVID-19 infection during the omicron surge in the US. The death rate due to COVID-19 was 9% (6/68) in the overall population, whereas the mortality rate was 26% (6/23) in hospitalized patients. During the preintervention COVID-19 era, the mortality rate in cancer patients from COVID-19 infection was 34%, and this increased in hospitalized patients to 60.2% [1,14]. Thus, the death rates in our study were much lower when compared to those in studies conducted in cancer patients earlier in the pandemic, and this may be attributed to modern interventions such as COVID-19 vaccinations and treatments. Furthermore, in our study, most patients (74%, 17/23) who were hospitalized or died due to COVID-19 infection did not receive COVID-19 mAb treatment, although this was not statistically significant and was likely due to low patient numbers. These results underscored the importance of humoral immunity in limiting disease severity and pointed to the protective effect of COVID-19 mAbs in improving outcomes in patients with immune dysfunction.

Early in the COVID-19 pandemic, it was hypothesized that immunocompromised patients would benefit from convalescent plasma therapy from donors who had recovered from COVID-19 infection [15]. In a study of 228 patients with severe COVID-19 infection, there was no significant difference in clinical status at day 30 in patients who received convalescent plasma compared to those who did not (odds ratio (OR) 0.83; 95% confidence interval (CI), 0.52 to 1.35; *p* = 0.46) [16]. Although initial studies failed to show clinical improvement following convalescent plasma administration, these studies included low numbers of patients with pre-existing malignancies or profound immunocompromisation [16]. Subsequent observational studies suggested that patients with hematologic malignancies, especially those receiving anti-CD20 monoclonal antibody treatment, may benefit from convalescent plasma infusions [15,17,18]. In addition, studies that evaluated the use of varying concentrations of neutralizing antibodies provided insight into the importance of using higher titers in patients with COVID-19 infection. A study that evaluated the use of convalescent plasma with high titers of neutralizing antibodies showed administration was safe, conferred transfer of antibodies, and improved the 30-day survival rate to 88.9% in non-mechanically ventilated patients when compared to an overall survival rate of 72% in a control group based on data from the entire Hackensack Meridian *Health* network [19]. Another study showed antibody titers significantly bolstered the therapeutic effect of convalescent plasma, with a reduction in the potential harmful effects of plasma with every log increase in neutralizing antibody titers (OR 0.74; 95% CI 0.57–0.95) [20]. Limitations of these trials included the use of different antibody titers and the timing of the administration of convalescent plasma. While these data may generate hypotheses, with the advent of anti-COVID-19 therapeutics, convalescent plasma has become obsolete.

A major advancement in the fight against COVID-19 was the development of the COVID-19 mRNA vaccines, which were found to confer 95% (Pfizer, BNT162b2 vaccine) and 94.1% (Moderna, mRNA-1273 vaccine) efficacy in preventing severe COVID-19 infection in the general population [6,7]. However, patients considered to be in an immunodeficient state or receiving immunosuppressive therapies were excluded from these landmark trials. In our study, there was no significant difference in COVID-19-related mortality or hospitalization between vaccinated and unvaccinated lymphoma patients. This finding was similar to those of other studies that evaluated the rates of breakthrough COVID-19 infections among cancer patients vaccinated against COVID-19. In a retrospective registry study of 6860 vaccinated patients, there was a higher rate of breakthrough COVID-19 infection among 1460 cancer patients when compared with noncancer patients (OR 1.12, 95% CI, 1.01 to 1.23; OR 4.64, 95% CI, 3.98 to 5.38, respectively) [21]. When adjusting for cancer types, hematologic malignancies were at increased risk for breakthrough infections compared to solid tumors (the adjusted OR ranged from 2.07 for lymphoma to 7.25 for lymphoid leukemia) [21]. Thus, patients with hematologic malignancies are particularly susceptible to breakthrough infection and subsequent severe COVID-19 infection [22].

It is possible that breakthrough infections in lymphoma patients are due to failed seroconversion. In our study, we had serology data available for 30 patients who received the COVID-19 vaccines and found 60% were not able to develop anti-COVID-19 spike protein antibodies. This increased risk may in part be due to intrinsic immune dysregulation as well as the cytotoxic and myelosuppressive effects of anticancer treatment. Moreover, the ability to mount an immune response against COVID-19 infection or seroconvert following COVID-19 vaccination may be dampened by impaired humoral immunity or depend on the type of therapy received. For instance, it is well known that patients diagnosed with B-cell NHLs who have undergone recent treatment with anti-CD20 monoclonal antibodies have further impaired serologic conversion, particularly if the time since the last anti-CD20 treatment was less than 6 months [23]. Although only 40% of vaccinated patients with serology data available in our study were able to seroconvert, we found that all (12/12) of these patients remained nonhospitalized during COVID-19 infection. Despite mutational characteristics of omicron that could potentially render antibody responses generated by COVID-19 vaccination ineffective, studies in healthy patients showed that CD4+ and CD8+ T-cell responses induced by COVID-19 vaccines were preserved at rates of approximately 84% against the omicron variant despite decreases in memory B cells and neutralizing antibodies [24,25]. In our patients, the higher-than-expected survival rate may have partly been due to this preserved mechanism of T-cell response despite diminished vaccine efficacy and impaired humoral recognition against omicron. Therefore, it still remains vitally important to vaccinate patients with lymphoma and CLL against COVID-19 because certain patients may derive benefit from vaccination if they are able to seroconvert. A study of 453 hematopoietic stem cell transplant patients showed that despite 32% of the patients not being able to seroconvert following the first dose of the COVID-19 vaccine, the patients were able to seroconvert with repeated or “booster” dosing. A 20-fold increase in titers from a booster dose compared to the primary vaccination series was observed and showed memory B cells in cancer patients were capable of an anamnestic immune response [26]. Additional studies are needed to better understand the benefit and scheduling of repeat COVID-19 vaccinations and the protective role of cellular immunity in cancer patients. 

Numerous mAb combinations, or “cocktails”, have been developed to target the spike protein of COVID-19 and block viral attachment with varying efficacy against COVID-19 variants. Initial trials evaluated the efficacy of mAb therapy in outpatients who were at risk for developing severe COVID-19 infection. In the BLAZE-1 trial, bamlanivimab/etesevimab significantly reduced the risk of COVID-19-related hospitalization or death from any cause in high-risk ambulatory patients (absolute risk difference, −4.8 percentage points; 95% CI, −7.4 to −2.3; relative risk difference, 70%; *p* < 0.001) [8]. Similarly, the RECOVERY trial evaluated casirivimab/imdevimab therapy and found a significant reduction in the 28-day mortality among patients who were seronegative at baseline [9]. The COMET-ICE study investigated sotrovimab use in nonhospitalized patients and found significantly fewer patients in the sotrovimab group had disease progression leading to hospitalization or death (relative risk reduction, 85%; 97.24%, 95% CI, 44 to 96; *p* = 0.002) [10]. Although these therapies have shown significant benefit in the general population, patients with malignancies were not included in these randomized controlled trials. 

With continued viral evolution, certain combination mAbs, including bamlanivimab/etesevimab and casirivimab/imdevimab, have lost their efficacy against novel variants, including omicron [27]. However, other COVID-19 mAbs, such as sotrovimab, bebtelovimab, and tixagevimab/cilgavimab (Evusheld), have retained their efficacy against the omicron variant [12]. Our findings were consistent with this, as there were fewer patients hospitalized from COVID-19 who received COVID-19 mAbs (26%, 6/23). Differently, 74% (17/23) of hospitalized patients did not receive mAb treatment for COVID-19 infection. Our results implied that COVID-19 mAb treatment is of critical importance to stimulate the humoral response against COVID-19 infection in lymphoma patients experiencing immune dysfunction due to their disease biology or receipt of immunosuppressive anticancer therapies. While COVID-19 mAbs have shown a significant benefit in the general population of patients at high risk for severe infection, there remains a gap in the literature regarding their use in patients with hematologic malignancies. In addition, the study design of landmark trials assessing the use of COVID-19 mAbs limits their clinical use only to those who develop COVID-19 infection while outpatient and can receive these therapies in the ambulatory setting during early infection, despite the theoretical benefit mAb therapy may provide an immunodeficient or immunosuppressed patient later in the course of COVID-19 infection or following hospital admission.

Similarly, the utility of antiviral agents in cancer patients with COVID-19 infection is unclear due to the prolonged duration of viral shedding and lack of data in this patient population [28]. Studies have shown that patients with hematologic malignancies remained positive for viral shedding 45 days after initially testing positive for COVID-19 infection, whereas immunocompetent patients rarely experienced live virus recovery after day 11 of illness [29]. Most antiviral agents indicated for COVID-19 treatment, such as remdesivir, molnupiravir, and nirmatrelvir/ritonavir (Paxlovid), are FDA-approved for 5- to 10-day courses of therapy, which may reduce the viral burden during treatment but increase the likelihood of rebound infection or resistance in patients with prolonged viral shedding [30,31,32]. More studies to further elucidate the role, optimal timing, and duration of COVID-19 treatments in cancer patients are needed. 

Limitations of this study included the retrospective, single-center design; the small study population; a lack of data regarding seropositivity in previously COVID-19 vaccinated patients; the low number of patients actively receiving anticancer treatment in the previous 6 months; a lack of patients receiving COVID-19 directed therapies other than mAb treatments; and a potential selection bias in patients who received COVID-19 mAbs who may have had mild-to-moderate infection. 

## 5. Conclusions

In conclusion, as the COVID-19 pandemic continues to evolve with novel VOCs, so does the complexity in managing patients particularly susceptible to severe disease, including those with hematologic malignancies. It is imperative to understand the COVID-19-related outcomes of patients with lymphoma and CLL so that medical management can be optimized accordingly.

Our study provided real-world data on lymphoma and CLL patients during the omicron surge in the US and confirmed that our patients experienced COVID-19 differently than the general population. Patients with lymphoma were still at risk for hospitalization and death from COVID-19, but these rates were comparatively lower when considering preintervention COVID-19. In this modern era of available therapeutics, prospective studies in oncology patients and COVID-19 guidelines specific to immunocompromised patients are needed in order to develop a different approach to utilizing available monoclonal antibody and antiviral treatments, as well as optimizing vaccination schedules to improved COVID-19 prevention and treatment in cancer patients.

## Figures and Tables

**Table 1:** Baseline characteristics.

Characteristics	Patients N = 68 *n* (%)
Age, median years (IQR)	66 (59–75)
Male	38 (56)
**Race**	
Black or African American	1 (2)
Hispanic or Latino	11 (16)
White	45 (66)
Other	11 (16)
**Disease type**	
Burkitt’s, DLBCL, PMBL	20 (29)
CLL/SLL	14 (21)
Hodgkin’s lymphoma (HL)	2 (3)
Indolent lymphomas ^†^	20 (29)
Mantle cell lymphoma (MCL)	3 (4)
T-cell lymphomas (TCLs) ^‡^	6 (10)
Other ^§^	3 (4)
**Comorbidities**	
Cardiovascular disease	23 (34)
COPD	5 (7)
Autoimmune disease ^††^	5 (7)
Mild liver disease ^‡‡^	2 (3)
Diabetes	19 (28)
Chronic kidney disease ^¶^	6 (10)
HIV/AIDS	1 (2)
Smoking history	
Current smoker	3 (4)
Former smoker	18 (27)
**Absolute neutrophil count at COVID-19 diagnosis**	
Median cells × 10^3^ (IQR)	3.25 (2.175–4.7)
Less than or equal to 1000 cells/mm^3^	6 (9)
**Absolute lymphocyte count at COVID-19 diagnosis**	
Median cells × 10^3^ (IQR)	1.1 (0.7–1.625)
Less than or equal to 500 cells/mm^3^	15 (22)
**Vaccine received**	
Pfizer-BioNTech	28 (41)
Moderna	24 (35)
Johnson & Johnson	1 (2)

DLBCL—diffuse large B-cell lymphoma; PMBL—primary mediastinal B-cell lymphoma; CLL—chronic lymphocytic leukemia; SLL—small lymphocytic lymphoma; COPD—chronic obstructive pulmonary disease; HIV—human immunodeficiency virus; AIDS—acquired immunodeficiency syndrome. ^†^ Indolent lymphomas included a diagnosis of one of the following: follicular lymphomas, marginal zone lymphoma, lymphoplasmacytic lymphoma, and Waldenstrom macroglobulinemia; ^‡^ T-cell lymphomas included a diagnosis of one of the following: peripheral T-cell lymphomas not otherwise specified (NOS), anaplastic large cell lymphoma (ALCL), cutaneous T-cell lymphomas, angioimmunoblastic T-cell lymphoma (AITL), and acute T-cell leukemia/lymphoma (ATLL); ^§^ included Langerhans histiocytosis, hemophagocytic lymphohistiocytosis (HLH), and T-cell large granular lymphocytic leukemia (T-LGL); ^¶^ cardiovascular disease was defined as history of coronary artery disease, hypertension, or cerebrovascular accident; ^††^ autoimmune disease included a diagnosis of one of the following: autoimmune disease, rheumatological disorders, and connective tissue disease; ^‡‡^ mild liver disease was defined as a history of chronic hepatitis or cirrhosis without portal hypertension.

**Table 2:** Primary outcomes.

Outcomes	Patients *n* (%)
**Hospitalization due to COVID-19 infection**	
Overall population	23/68 (34)
**Death due to COVID-19 infection**	
Overall population	6/68 (9)
Hospitalized patients	6/23 (26)

**Table 3:** Secondary outcomes.

Patient Subgroups	COVID-19-Associated Hospitalization or Death		*p*-Value; UVA OR (95% CI), *p*-Value; MVA OR (95% CI), *p*-Value
	Yes n (%)	No n (%)	
COVID-19 vaccine received *			*p* = 0.759; 0.729 (0.223, 2.382), *p* = 0.6; 0.929 (0.222, 3.886), *p* = 0.919
Yes (n = 52)	17/23 (74)	35/45 (78)	
No (n = 15)	6/23 (26)	9/45 (20)	
**COVID-19 mAb treatment received**			*p* = 0.070; 0.338 (0.112, 1.013), *p* = 0.053; 0.324 (0.096, 1.098), *p* = 0.07
Yes (n = 29)	6/23 (26)	23/45 (51)	
No (n = 39)	17/23 (74)	22/45 (49)	
**Anti-CD20 mAb or BTKi-containing regimen ^φ^**			*p* = 0.907 1.5 (0.386, 5.825), *p* = 0.558; 2.899 (0.459, 18.319), *p* = 0.258 1 (0.15, 6.671), *p* = 1; 1.492 (0.178, 12.489), *p* = 0.712 0.857 (0.239, 3.079), *p* = 0.813; 1.571 (0.281, 8.775), *p* = 0.607
Anti-CD20 mAb monotherapy (n = 14)	6/23 (26)	8/45 (18)	
BTKi monotherapy (n = 6)	2/23 (9)	4/45 (9)	
Anti-CD20 mAb + chemotherapy (n = 20)	6/23 (26)	14/45 (31)	
All others (ref) (n = 25)	8/23 (35)	17/45 (38)	
**Lymphoma diagnosis**			*p* = 1 1.148 (0.327, 4.028), *p* = 0.829, 1.789 (0.321, 9.961), *p* = 0.507 1.240 (0.261, 5.891), *p* = 0.787; 1.157 (0.167,8.03), *p* = 0.883
CLL/SLL (n = 14)	5/23 (22)	9/45 (20)	
HL/TCL (n = 8)	3/23 (13)	5/45 (11)	
All others (ref) (n = 46)	15/23 (65)	31/45 (69)	
**Anticancer treatment in previous 6 months**			*p* = 0.481 1.368 (0.379, 4.944), *p* = 0.632; 0.725 (0.143, 3.68), *p* = 0.68976 0.625 (0.144, 2.718), *p* = 0.531; 0.366 (0.063, 2.12), *p* = 0.2622
Yes (n = 32)	13/23 (57)	19/45 (42)	
No (n = 21)	5/23 (9)	16/45 (36)	
All others (ref) (n = 15)	5/23 (9)	10/45 (22)	

UVA—univariate analysis; OR—odds ratio; CI—confidence interval; MVA—multivariate analysis; mAb—monoclonal antibody; BTKi—Bruton’s tyrosine kinase inhibitor; CLL—chronic lymphocytic leukemia; SLL—small lymphocytic lymphoma; HL—Hodgkin’s lymphoma; TCL—T-cell lymphomas. * One patient with unknown COVID-19 vaccination status; **^φ^** 3 patients with unknown treatment history with an anti-CD20 mAb or BTKi.

## Data Availability

The data presented in this study are available upon request from the corresponding author. The data are not publicly available because this study involved a review of the electronic medical records of lymphoma patients who developed COVID-19 infection. Most, but not all, of the protected health information was removed from the research dataset (for example: the actual dates of COVID-19 PCR assays were included as needed for analysis but would be considered PHI). The dataset is also considered property of Hackensack Meridian *Health* and is not owned by the investigators. Therefore, for both reasons, the dataset cannot be made openly available. However, upon request, we are willing to share portions of the data for appropriate review. Requests can be made through the corresponding author or directly to representatives of Hackensack Meridian *Health* (Tatyana A. Feldman; email: tatyana.feldman@hmhn.org).
